# Membrane Elastic Properties and Cell Function

**DOI:** 10.1371/journal.pone.0067708

**Published:** 2013-07-03

**Authors:** Bruno Pontes, Yareni Ayala, Anna Carolina C. Fonseca, Luciana F. Romão, Racκele F. Amaral, Leonardo T. Salgado, Flavia R. Lima, Marcos Farina, Nathan B. Viana, Vivaldo Moura-Neto, H. Moysés Nussenzveig

**Affiliations:** 1 LPO-COPEA, Instituto de Ciências Biomédicas, Universidade Federal do Rio de Janeiro, Rio de Janeiro, Rio de Janeiro, Brazil; 2 Instituto de Física, Universidade Federal do Rio de Janeiro, Rio de Janeiro, Rio de Janeiro, Brazil; 3 Universidade Federal do Rio de Janeiro/Macaé, Macaé, Rio de Janeiro, Brazil; 4 Diretoria de Pesquisas, Instituto de Pesquisas Jardim Botânico do Rio de Janeiro, Rio de Janeiro, Rio de Janeiro, Brazil; University of the Witwatersrand, South Africa

## Abstract

Recent studies indicate that the cell membrane, interacting with its attached cytoskeleton, is an important regulator of cell function, exerting and responding to forces. We investigate this relationship by looking for connections between cell membrane elastic properties, especially surface tension and bending modulus, and cell function. Those properties are measured by pulling tethers from the cell membrane with optical tweezers. Their values are determined for all major cell types of the central nervous system, as well as for macrophage. Astrocytes and glioblastoma cells, which are considerably more dynamic than neurons, have substantially larger surface tensions. Resting microglia, which continually scan their environment through motility and protrusions, have the highest elastic constants, with values similar to those for resting macrophage. For both microglia and macrophage, we find a sharp softening of bending modulus between their resting and activated forms, which is very advantageous for their acquisition of phagocytic functions upon activation. We also determine the elastic constants of pure cell membrane, with no attached cytoskeleton. For all cell types, the presence of F-actin within tethers, contrary to conventional wisdom, is confirmed. Our findings suggest the existence of a close connection between membrane elastic constants and cell function.

## Introduction

The cell membrane with its associated proteins, besides enclosing the cell internal environment and controlling endocytosis and exocytosis, takes part in a variety of vital processes, including molecule presentation and recognition, catalysis, signal sensing, cytokinesis, cell shaping and motility. Through its interaction with the cytoskeleton and motor proteins, it exerts and responds to forces [1], [2].

The elastic properties of the cell membrane, specially its bending modulus 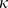 [3] and surface tension σ [4], are basic parameters underlying the dynamics of these processes. Present data on cell specialization and differentiation allow us to conjecture the possible existence of correlations between the values of these parameters and specialized cell functions associated with shape deformation and/or force production, as exemplified by phagocytosis [5].

In the present work, we test this conjecture by measuring 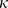 and σ for a variety of cell types, especially brain cells. Our measurement technique is based on tether extraction from the cell by pulling on it with an attached microsphere trapped by optical tweezers [6], [7], [8]. Analysis of the force-extension curve, together with measurement of the tether radius, yields the values of the elastic parameters and information on the membrane-cytoskeleton interaction [9]. Comparative analysis of our results is compatible with the above conjecture. Indeed, we find not only that cells with phagocytic functions have differentiated membrane elastic properties, but also that such properties undergo sharp changes between their quiescent and activated forms.

The central nervous system is a good candidate to analyze the role of force production and/or shape deformation in cell function, since forces, displacements and deformations play a significant role in neural cell activity. Neurons are highly anisotropic cells, with relatively quiescent cell bodies but dynamic axons and dendrites, susceptible to large structural changes in dendritic branches [10]. Astrocytes are remarkably dynamic, constantly modifying their morphology during migration [11]. Glioblastoma cells migrate long distances to invade brain regions [12], [13], [14]. Macrophages, besides their motility, undergo actin remodeling and strong deformations during phagocytosis [15], [16]. Microglia, the professional phagocytes of the brain, continually scan their environment through motility and protrusions [17], [18], [19], [20].

In order to assess the relative roles of membrane elasticity and of its interaction with the attached cytoskeleton in cell specializations, we also undertake to measure the elastic properties of “pure cell membrane”, a cell membrane detached from the cytoskeleton. In a normal eukaryotic cell, the membrane is supported by the cell cortex, an F-actin scaffolding [21]. The cortex is ruptured when a bleb gets formed, and it remains absent during bleb expansion, that typically takes ∼30 s, followed by cortex regrowth and bleb retraction, lasting ∼2 min and powered by myosin II [22]. Force measurements taken during the rapid bleb expansion, therefore, could be contaminated by friction between the two leaflets of a plasma membrane, which may produce substantial effects [23], [24], [25].

To avoid such problems, we prefer to extract tethers from PMV – plasma membrane vesicles [26]. PMV’s are vesicles formed upon exposure of cells to low concentrations of specific reagents, see Materials and Methods for details [27]. They are supposed to be formed by weakening the membrane-cytoskeleton connection followed by pressure-driven volume expansion [28] and to have the general characteristics of plasma membranes [29].

The results are consistent with other approximations to “pure cell membrane”, based on progressive disruption of the cytoskeleton [9]. A critical appraisal of the applicability of the theoretical model employed for the determination of the elastic constants is given.

## Materials and Methods

### Animals

This study was approved by the Ethics Committee of the Health Sciences Center, Federal University of Rio de Janeiro (Protocol No. DAHEICB 015). The “Principles of laboratory animal care” (NIH publication No. 85–23, revised 1996) guidelines were strictly followed for all experiments. Swiss mice were obtained from the Biomedical Sciences Institute, Federal University of Rio de Janeiro.

### Cell Cultures

Seven different cell types were used in this work. Two of them were glioblastoma cell lineages, U-87 MG and GBM95 [30]. The other five were primary cultures obtained from Swiss mice: cortical neurons (Neurons CX), ganglionic eminence neurons (Neurons GE), cortical astrocytes, microglia and peritoneal macrophages.

The glioblastoma cell lineages were cultured in Dulbecco’s modified Eagle’s medium (DMEM-F12) containing L-glutamine, 10% fetal bovine serum and 1% penicillin/streptomycin. All culture reagents, unless otherwise noted, were purchased from Invitrogen (Carlsbad, CA, USA).

The neurons primary cultures were obtained from two different regions of E14 Swiss mouse embryos, following previously established procedures [31], [32], [33]. Briefly, neuron cell suspensions were obtained by dissociating the cells from the cerebral cortex or ganglionic eminence and plated in Neurobasal media supplemented with L-glutamine, 1% penicillin/streptomycin and 2% of B27 the day before the experiments.

The cortical astrocytes and microglial primary cell cultures were both obtained from neonatal Swiss mice following procedures from [31], [34], [35], [36]. Briefly, astrocytes primary cultures were prepared from cell suspensions obtained by dissociating the cerebral cortex. Cells were plated in DMEM-F12 medium with L-glutamine, 10% fetal bovine serum and 1% penicillin/streptomycin. Cells were allowed to proliferate until reaching confluence. Floating microglial cells were isolated from two-week primary astrocytes cultures that were grown as described. Isolated microglial cells presented above 99% purity. Two different types of microglial cells were used, one non-activated, referred to as control microglia, and another one previously treated with 1 µg.mL^−1^ of LPS (Lipopolysaccharide, a polysaccharide that elicits strong immune responses; Sigma-Aldrich, St. Louis, MO, USA) for 24 hours, an activated microglial cell, referred to as microglia+LPS.

Peritoneal macrophages were obtained from adult Swiss mice by an i.p. injection with 2 ml phosphate-buffered saline (PBS). After 5 min, peritoneal macrophages were collected, washed three times with cold PBS, and then plated, the day before the experiments. Two different types of macrophage cells were used, one non-activated, referred to as control macrophage, and another one activated by previous treatment with 10 µg.mL^−1^ of LPS for 24 hours, referred to as macrophage +LPS.

With the exception of neurons and macrophages, whose cultures were prepared and plated one day before the experiments, all other cells used in this work were maintained at 37°C and 5% CO_2_ until they reached confluence and then were re-plated the day before the experiments. 5 × 10^4^ cells of each type were plated on an 18×18 mm glass coverslip pre-coated with poly-L-lysine and placed within a special 35 mm glass bottom culture dish.

### Optical Tweezers Setup

The optical tweezers (OT) system used in this work employs an infrared Nd:YVO4 Osprey laser with a wavelength of 1.064 µm (Quantronix, East Setauket, NY, USA). The laser has a Gaussian intensity profile (TEM_00_ mode), with power at the sample below 400 mW and beam half width of 2.3±0.2 mm at the back focal plane of the objective lens. The infrared laser system couples with an inverted Nikon Eclipse TE300 microscope (Nikon, Melville, NY, USA), equipped with a PLAN APO 100X 1.4 NA DIC H Nikon objective, used to create the optical trap.

### Trap Calibration and Force Measurements

To calibrate the OT, we followed the procedures described in [9]. Briefly, a polystyrene bead with radius *a* = 1.52±0.02 µm (Polysciences, Warrington, PA, USA) was trapped and the sample was set to move with velocity *V*. Movies of the entire process were collected with a CCD Hamamatsu C2400 camera (Hamamatsu, Japan) connected with a SCION FG7 frame grabber (Scion Corporation, Torrance, CA, USA). The radial position of the trapped bead, ρ, was obtained by image analysis using Image J software (National Institutes of Health, Bethesda, MD, USA). The displacement Δ
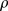
is defined as the difference between the bead position when the bead is moving with velocity *V* and its position when the bead is not moving:
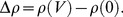
(1)


For small transverse displacements of the bead in the trap (∼1 µm), we have:
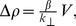
(2)where 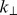 is the transverse trap stiffness (perpendicular to the beam direction of propagation) and β is the Stokes fluid friction coefficient. By moving the sample with different values of *V* and measuring the bead position displacement, the trap stiffness 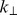
is obtained. The trap stiffness values can be increased or decreased in proportion to the laser beam power [9].

Using the trap calibration, the displacement of a trapped bead in relation to its trap equilibrium position multiplied by the value of the trap stiffness gives the force on the bead:
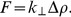
(3)


### Tethers Extracted from Cells

Tether extraction experiments were performed following the same procedures as described in [9]. Briefly, 5 × 10^4^ cells of each of the cell types used in this work were plated on glass bottom dishes, as described above. 24 hours after plating, uncoated polystyrene beads (radius *a* 1.52±0.02 µm) were added and each of the glass bottom dishes was placed in the OT microscope. The OT was used to trap a bead and to press it against the chosen cell membrane for 5 seconds, allowing the beads to attach to the cell. Bead attachment is likely to be mediated by van der Waals and other short-range forces. As was demonstrated in [9], bead treatment with a variety of coatings does not affect the results. Then, the microscope motorized stage (Prior Scientific, Rockland, MA, USA) was set to move with a controlled velocity *V* = 1 µm/s. Movies of the entire process were collected by a CCD Hamamatsu C2400 camera (Hamamatsu, Japan) connected with a SCION FG7 frame grabber (Scion Corporation, Torrance, CA, USA), using a capture frame rate of 10 frames/second. Using the trap calibration described above, the measured bead position displacement was converted into measured force. All the OT experiments were performed in a CO_2_ chamber adapted to the microscope, maintaining optimal culture conditions during the experiments (37°C and 5% CO_2_ pressure). All the data analysis and force calculations described were performed with Kaleidagraph software (Synergy Software, Essex Junction, VT, USA).

### Fluorescence Microscopy

Immunofluorescence was performed for each of the cell types used in order to verify their purity. Briefly, they were fixed in PBS-paraformaldehyde 4% for 15 min, treated with PBS-triton X100 0.2% for 5 min, blocked with PBS+5% BSA (Sigma-Aldrich, St. Louis, MO, USA) for 1 hour at 37°C and then incubated overnight at 4°C with primary antibodies for each of the specific markers: for neurons, monoclonal antibody against the β-tubulin III protein (Promega Corporation, Madison, WI, USA); for astrocytes, polyclonal antibody against glial fibrillary acidic protein (GFAP, a specific astrocyte marker) (Dako, Denmark); for microglial cells, monoclonal antibody against F4/80 protein (AbD Serotec, Raleigh, NC, USA) and for macrophage cells, monoclonal antibody against CD68 protein (AbD Serotec, Raleigh, NC, USA). Then, secondary monoclonal or polyclonal Alexa 546nm antibodies (Molecular Probes Inc, Eugene, OR, USA) were incubated for 1 hour as fluorescent probes. Finally, all cells were also stained for 1 hour with phalloidin-FITC (Sigma-Aldrich, St. Louis, MO, USA) and were visualized using a Leica TCS-SP5 II confocal microscope (Leica Microsystems, Germany). Confocal fluorescence images were captured employing LAS AF 2.2.0 Software (Leica Microsystems, Germany).

### Radius Measurements of Tethers Extracted from Cells

5 × 10^4^ cells of each of the cell types used in this work were plated in glass bottom dishes 24 hours before the experiment. The same procedures to extract tethers from the cell surfaces were performed as described above. Immediately after the tether pulling experiments, cells were fixed and prepared for scanning electron microscopy, using the same protocol established before [9]. After image acquisition and analysis, tether radius measurements for each cell type were performed following the same procedures described before [9].

### Determination of Surface Tension and Bending Modulus

**Figure 1:**
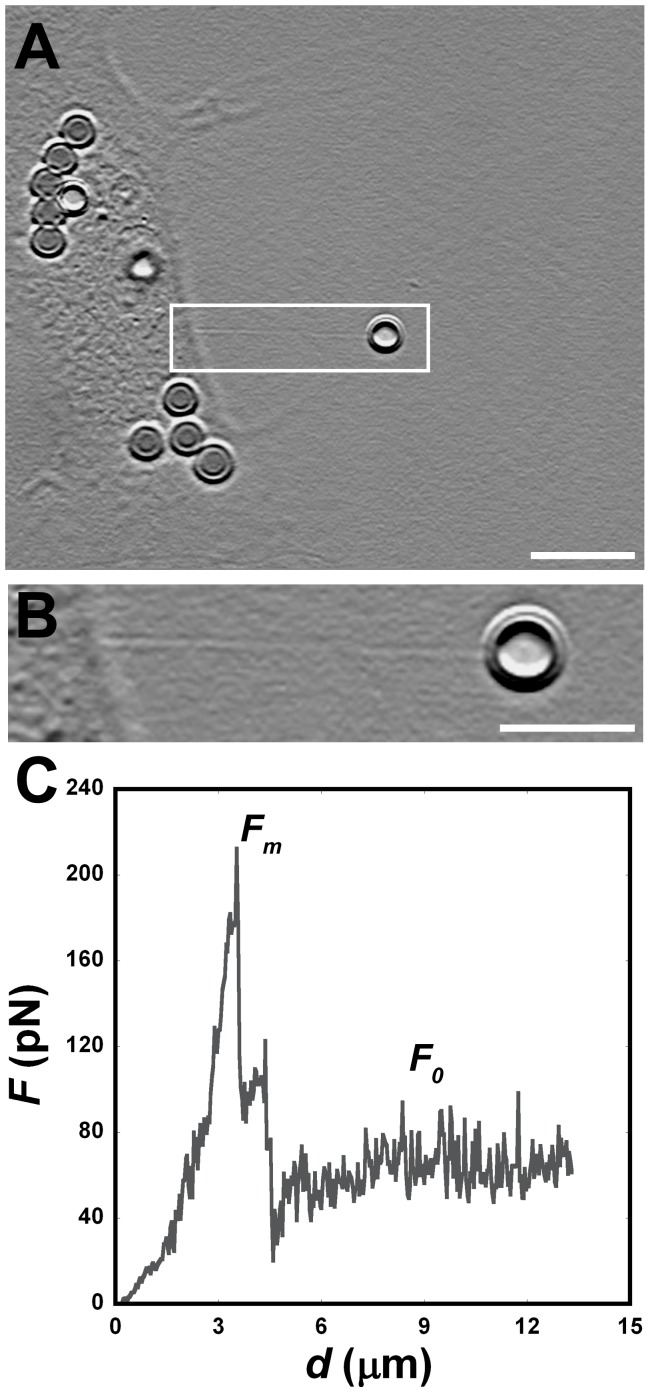
Tether extraction from microglial cell. (A) Image of the extracted tether in bright field with Image J shadow north processing filter applied. (B) Zoom of the white rectangle in A. Scale bar for A is 10 µm and for B is 5 µm. (C) A typical tether extraction force curve, indicating the maximum force *F_m_* and the approach to *F*_0_, the steady-state tether force.

According to the theories of tether extraction [37], [38], based on free energy minimization [39], the membrane elastic constants are related to the tether radius *R* and steady-state tether force *F*_0_ (cf. Fig. 1) by 
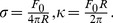
(4)From the measured values of *F*_0_ and *R*, we get σ and 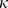
by applying Eq. (4).

### Plasma Membrane Vesicles Induction Experiments

To induce the formation of plasma membrane vesicles (PMV) on the surface of cells, for all the cell types used in this work, we employed a solution previously described in the literature [26]. Briefly, after plating and before the OT experiments, each one of the cell types was incubated with this solution, containing 25 mM of formaldehyde, 20 mM of dithiothreitol (DTT), 2 mM of calcium chloride (CaCl2), 10 mM of HEPES, 0.15 M of sodium chloride (NaCl) and pH 7.4.

Cells were incubated in this solution for at least 30 min in order to form PMV. Then, the solution was removed, the culture medium was returned to each of the cell cultures and the experiments were performed.

### Tethers Extracted from PMV

After the PMV induction, cells of each of the cell types used in this work were placed in the OT microscope with uncoated polystyrene beads (radius *a* = 1.52±0.02 µm). The OT was used to trap a bead and to press it against a chosen cell PMV for 5 seconds, allowing the bead to attach to its surface. Then, the microscope motorized stage (Prior Scientific, Rockland, MA, USA) was set to move with a controlled velocity *V* = 1 µm/s. Movies of the entire process were collected by a CCD Hamamatsu C2400 camera (Hamamatsu, Japan) connected to a SCION FG7 frame grabber (Scion Corporation, Torrance, CA, USA), with a capture frame rate of 10 frames/second. Using the trap calibration described above, the measured bead position displacement was converted to measured force. All the OT experiments were performed in a CO_2_ chamber adapted to the microscope, maintaining optimal culture conditions during the experiments (37°C and 5% CO_2_ pressure). All the data analysis and force calculations described were performed with Kaleidagraph software (Synergy Software, Essex Junction, VT, USA).

### Measurements of Radius of Tethers Extracted from PMV

The protocol described above to measure the tether radius for tethers extracted from cells employs fixation procedures for scanning electron microscopy. However, it was not possible to apply this procedure to tethers extracted from PMV, because they were very fragile against all the steps. Therefore, a new method was adopted.

The new method is based upon the force barrier theory for tether formation, previously demonstrated [40] and supported by recent work, which reconfirmed its reliability [9]. This theory takes into account the fact that the contact area between the trapped bead and the cell membrane is a circular patch of radius 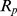 rather than a point, as had been assumed in previous treatments [37], [38]. This increases the maximum force 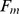 that the cell membrane can support, beyond which a sharp shape transition (cf. Fig. 1) leads to tether formation, yielding
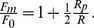
(5)where 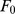 is the steady-state tether force and *R* is the tether radius.

Since we have 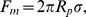 as follows from the definitions of 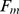 and of surface tension, and 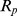 is >>*R* (cf. Table 1), Eq. (5) implies 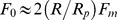, which yields 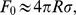 in agreement with Eq. (4). The reason for the extra factor of 2 is the role of bending stiffness in nanotube formation. If only surface tension were present, free energy would be minimized by *R* = 0 (infinitely thin filaments), but this would correspond to infinite curvature. The steady-state value of the nanotube radius *R* results from a compromise between surface tension and bending energy.

**Table 1:** Surface tension and bending modulus values.

Cell Types	(pN)	*R* (nm)	σ (10^–5^ N/m)	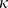 (10^–19^ J)
Astrocyte	32±4	51±2	5.0±0.7	2.6±0.3
GBM95	32±4	51±3	5.1±0.6	2.8±0.3
U-87 MG	33±3	48±4	5.3±0.8	2.5±0.4
Neuron CX (Cell Body)	16±2	78±5	1.6±0.2	1.9±0.2
Neuron CX (Neurite)	14±2	80±4	1.5±0.2	1.8±0.2
Neuron CX (Growth Cone)	15±2	75±7	1.6±0.2	1.8±0.3
Neuron GE (Cell Body)	17±2	87±6	1.6±0.2	2.3±0.3
Neuron GE (Neurite)	16±3	76±6	1.6±0.3	2.0±0.4
Neuron GE (Growth Cone)	15±2	77±7	1.5±0.3	1.8±0.3
Control Microglia	63±6	88±6	5.6±0.7	8.7±1.0
Microglia +1 µg.mL^−1^ LPS	41±3	43±2	7.8±0.6	2.8±0.2
Control Macrophage	68±5	72±7	7.5±0.9	7.8±1.0
Macrophage +1 µg.mL^−1^ LPS	69±4	73±6	7.5±0.7	8.0±0.8
Macrophage +10 µg.mL^−1^ LPS	32±2	41±3	6.2±0.6	2.1±0.2
PMVs	16.0±0.3	158±6	0.80±0.03	4.1±0.2

The values of 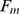 and 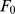 were experimentally determined in the force curve; 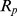 was measured from the image of the bead and the deformed cell surface, as described before [9]. This allows us to obtain the value of *R* by applying Eq. (5) (see the Results Section for details).

### Statistical Analysis

All data are presented as (mean ± standard error). Data were analyzed using GraphPad Prism statistics software (GraphPad Software, Inc. La Jolla, CA, USA). Student’s t-test or one-way ANOVA were used to compare the measured values described. A value of p<0.05 was considered significant.

## Results

### Tether Extraction Experiments

Images associated with a tether extraction experiment from a microglial cell are shown in Fig. 1: the extracted tether attached to the trapped microsphere appears in Figs. 1A and 1B. The corresponding tether extraction force *F* as a function of the pulling distance *d* is displayed in Fig. 1C, showing the rising portion, from the origin to the maximum; the maximum force, 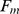; and a sharp drop to a much lower value 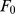, referred to as the (steady-state) tether force.

### Tether Extraction Experiments and Radius Measurements for Neurons

**Figure 2:**
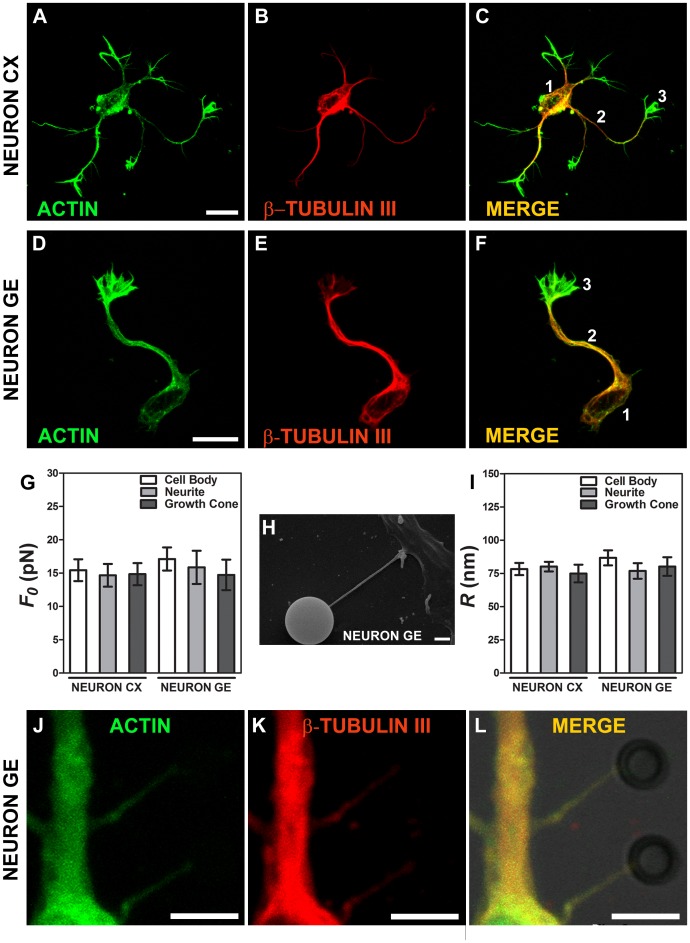
Tether extraction and radius measurements for neurons. (A–C) Images of the cortical (CX) neurons cytoskeleton stained for F-actin with phaloidin-FITC, in green (A), β-tubulin III, in red (B) and the merge of both images (C). (D–F) Images of the ganglionic eminence (GE) neurons cytoskeleton stained for F-actin with phaloidin-FITC, in green (D), β-tubulin III, in red (E) and the merge of both images (F). (C) and (F) also display numbers locating the 3 different regions from which tethers were extracted, (1) cell body; (2) neurite; (3) growth cone. (G) Mean values of the tether force, 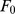, extracted from both Neuron CX and Neuron GE in regions 1, 2 and 3. (H) SEM image of a typical tether extracted from Neuron GE. Scale bar is 1 µm. (I) Mean values of the tether radius, *R*, extracted from both Neuron CX and Neuron GE in regions 1, 2 and 3. Standard errors were used as error bars in (G) and (H). At least 20 different experiments were performed for each situation in (G) and (H). (J)–(L) Images of tethers extracted from Neuron GE stained for F-actin with phaloidin-FITC, in green (J), β-tubulin III, in red (K) and the merge of both images (L). Scale bar is 5 µm.

Cultured neurons from two different cerebral regions were used in this work, neurons obtained from the cortex region (Neuron CX), and neurons from the ganglionic eminence region (Neuron GE). In Figs. 2A–F we display typical neurons from both regions stained in green, for phaloidin-FITC (a molecule commonly used as a cytochemical marker of polymerized actin) (Figs. 2A and 2D), and in red, for β-tubulin III, a neuronal marker (Figs. 2B and 2E). Merged figures are shown in Figs. 2C and 2F. Since neurons are cells with very peculiar anisotropic morphology, showing three different regions, we decided to perform tether extraction measurements on each of these different locations: the cell body (Figs. 2C and 2F (location 1)), neurite (Figs. 2C and 2F (location 2)) and growth cone (Figs. 2C and 2F (location 3)).

Mean values of the tether force, 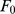, for each of the regions of the neuron cells are shown in Fig. 2G. We did not observe any statistically significant difference in tether force values among these regions. The values found were on the order of 15 pN of force. We also measured the tether radius for both neurons in each of the different regions. Fig. 2H is an example of a scanning electron microscopy (SEM) image showing the tether extracted from a neuron GE. Fig. 2I shows the mean values measured for the tether radius of each of the cells and each of the regions. We also did not observe any statistically significant difference among these values. The results found were on the order of 75 nm of radius.

Figs. 2J-L show images of tethers extracted from neurons GE stained for F-actin with phaloidin-FITC, in green (Fig. 2J), β-tubulin III, in red (Fig. 2K) and the merge of both images (Fig. 2L). The presence of both F-actin and β-tubulin III within the tethers is verified.

### Tether Extraction Experiments and Radius Measurements for Microglial Cells

**Figure 3:**
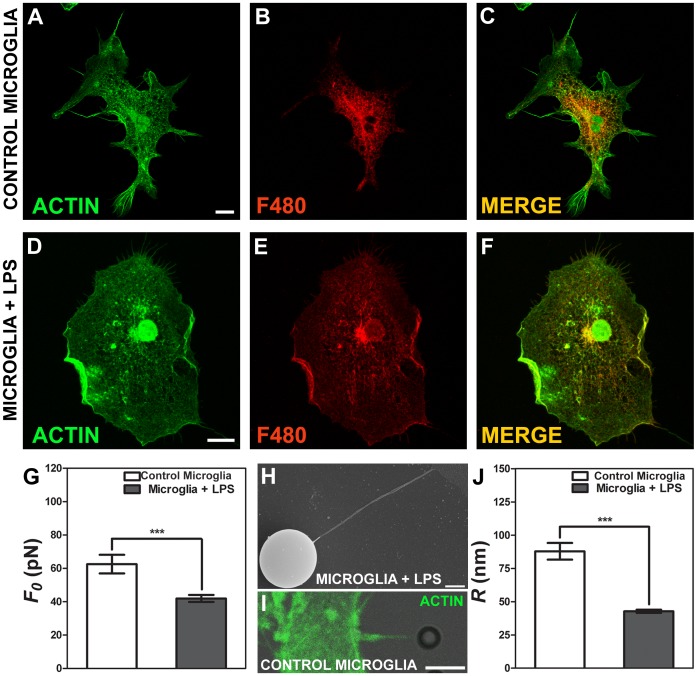
Tether extraction and radius measurements for microglial cells. (A–C) Images of the control microglia cytoskeleton stained for F-actin with phaloidin-FITC, in green (A), stained with F480, in red (B) and both images merged (C). (D–F) Images of the microglia+LPS cytoskeleton stained for F-actin with phaloidin-FITC, in green (C), F4/80, in red (D) and both images merged (F). Scale bars for A–F are all 10 µm. (G) Mean values of the tether force, 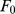, extracted from microglial cells. (H) SEM image of a typical tether extracted from microglia+LPS. Scale bar is 1 µm. (I) Image of tether extracted from control microglia stained for F-actin with phaloidin-FITC, in green. Scale bar is 5 µm. (J) Mean values of the tether radius, *R*, extracted from both microglial cell conditions. Standard errors were used as error bars in (G) and (J). At least 20 different experiments were performed for each situation in (G) and (J) (*** means p<0.0001 in t-test statistics).

Two types of microglial cells were used in this work, a non-activated microglial cell, that was called control microglia and a 1 µg.mL^−1^ LPS activated microglial cell, that was called microglia+LPS. In Fig. 3A–F we demonstrate typical microglial cells stained for phaloidin-FITC, in green, (Figs. 3A and 3D), and for F4/80 (a microglial marker), in red, (Figs 3B and 3E). Figs. 3C and 3F show the anterior ones merged. Values of the tether force,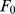, for each microglial cell condition are shown in Fig. 3G. We observed a statistically significant difference in tether force values (p<0.0001 in t-test). The values found for control microglia were 1.5 times larger than the values for microglia+LPS. We also measured the tether radius for both microglial cell conditions. Fig. 3H is an example of a SEM image showing the tether extracted from an activated microglia (microglia+LPS). Fig. 3I is an image of a tether extracted from control microglia, stained for F-actin with phaloidin-FITC, showing that F-actin is present in the tether. Fig. 3J shows the mean values measured for the tether radius of each of the cells. We observed a statistically significant difference for the values obtained (p<0.0001 in t-test): the values for control microglia were 1.8 times larger than those for microglia+LPS.

### Tether Extraction Experiments and Radius Measurements for Macrophage Cells

**Figure 4:**
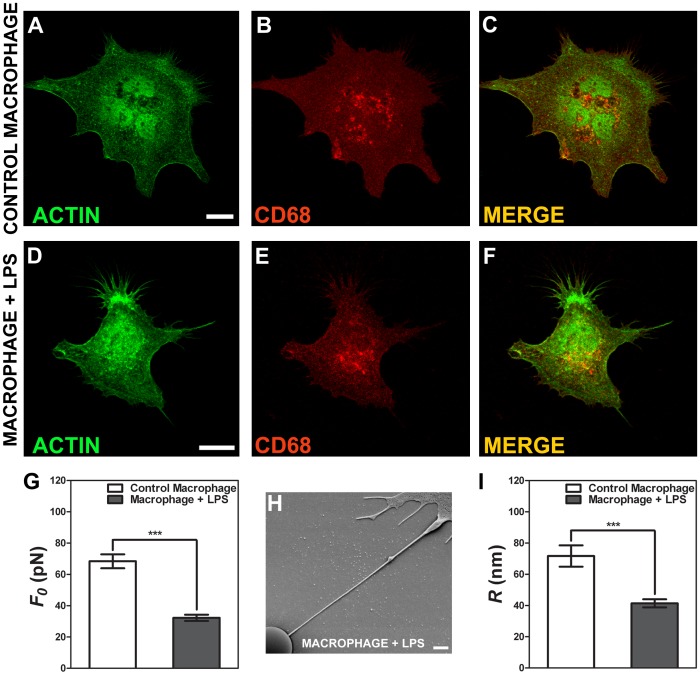
Tether extraction and radius measurements for macrophage cells. (A–C) Images of the control macrophage cytoskeleton stained for F-actin with phaloidin-FITC, in green (A), stained with CD68, in red (B) and both images merged (C). (D–F) Images of the macrophage+LPS cytoskeleton stained for F-actin with phaloidin-FITC, in green (C), CD68, in red (D) and both images merged (F). Scale bars for A-F are all 10 µm. (G) Mean values of the tether force, 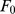, extracted from macrophage cells in both conditions. (H) SEM image of a typical tether extracted from macrophage+LPS cells. Scale bar is 1 µm. (I) Mean values of the tether radius, *R*, extracted from macrophage cells in both conditions. Standard errors were used as error bars in (G) and (I). At least 20 different experiments were performed for each situation in (G) and (I). (*** means p<0.0001 in t-test statistics).

Two types of macrophages were used, a non-activated macrophage cell, called control macrophage, and a 10 µg.mL^−1^ LPS activated macrophage cell, called macrophage+LPS. A concentration of 1 µg.mL^−1^ of LPS was initially tested, but no significant differences were observed (p>0.05 in t-test) for this cell type when compared with control (see Table 1). Based on previous works in the literature [41], we increased the LPS concentration to 10 µg.mL^−1^ to activate the macrophages. In Fig. 4A-F we represented typical macrophage cells stained for phaloidin-FITC, in green, (Figs. 4A and 4D), and for CD68 (a specific macrophage marker), in red (Figs 4B and 4E). In Figs. 4C and 4F the anterior ones are merged. Values of the tether force, 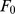, for each macrophage type are shown in Fig. 4G. We observed a statistically significant difference in tether force values. The values found for control macrophage are 2.0 times larger than the values found for macrophage+LPS (p<0.0001 in t-test). We also measured the tether radius for both macrophage cell conditions. Fig. 4H is an example of a SEM image showing a tether extracted from an activated macrophage (macrophage+LPS). Fig. 4I shows the mean values measured for the tether radius of each of the cells. We also observed a statistically significant difference in radius (p<0.0001 in t-test). The values found for control macrophage are almost twice as large as those for macrophage+ LPS.

### Tether Extraction Experiments and Radius Measurements for Astrocytes and Glioblastoma Cells

**Figure 5:**
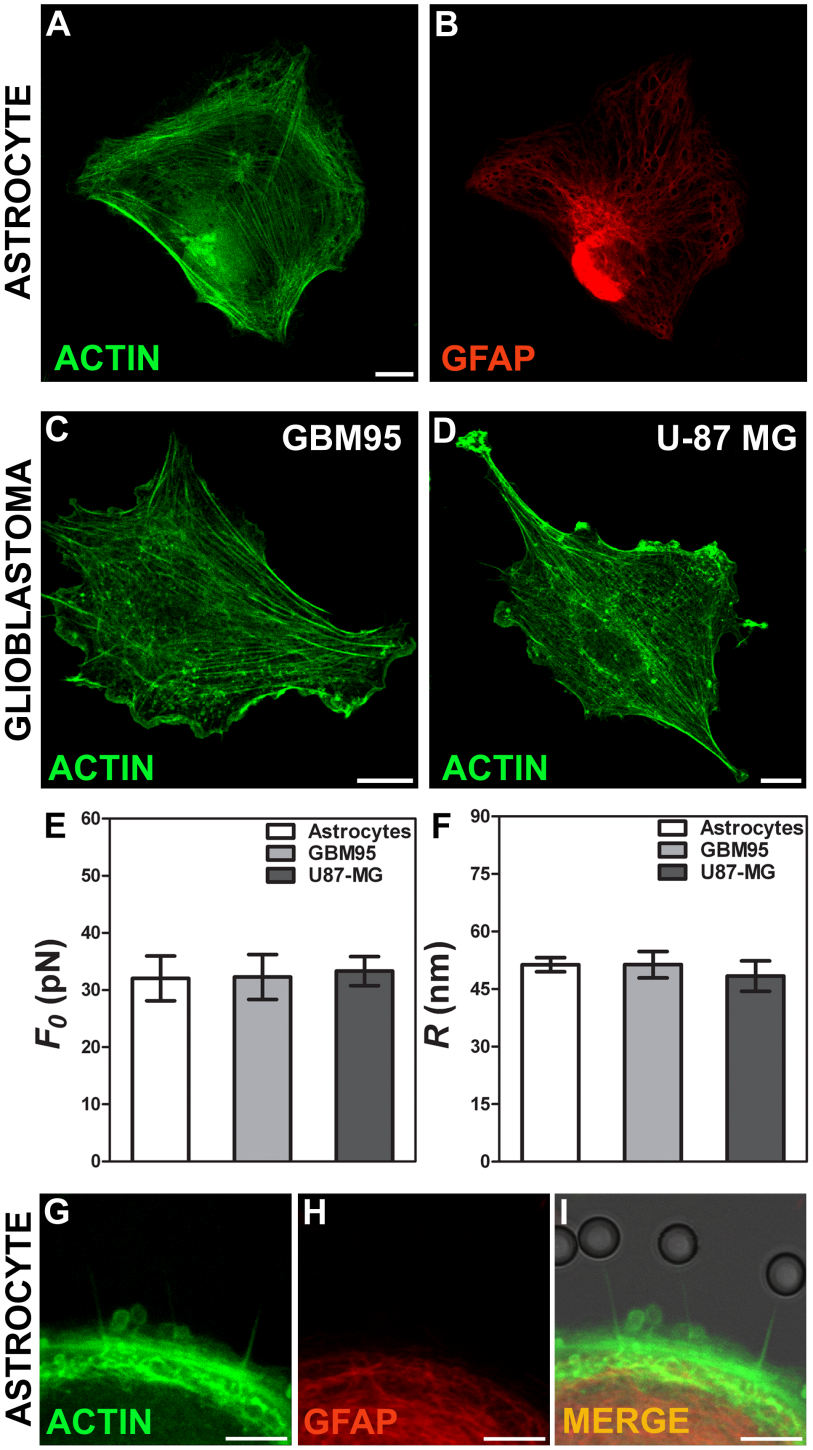
Tether extraction and radius measurements for astrocytes and glioblastoma cells. (A-B) Images of the astrocyte cytoskeleton stained for F-actin with phaloidin-FITC, in green (A) and GFAP, in red (B). (C-D) Images of glioblastomas U-87 MG and GBM95 cytoskeleton, respectively stained for F-actin with phaloidin-FITC, in green. Scale bars for A-D are all 10 µm. (E) Mean values of the tether force, 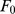, extracted from astrocytes and glioblastomas cells. (F) Mean values of the tether radius,*R*, extracted from astrocytes and glioblastomas cells. Standard errors were used as error bars in (E) and (F). At least 20 different experiments were performed for each situation in (E) and (F). (G-I) Images of tethers extracted from astrocytes, stained for F-actin with phaloidin-FITC, in green (G) and GFAP, in red (H). (G) and (H) merged in (I). Scale bars for G-I are all 5 µm.

Two types of glioblastoma cells, U-87 MG and GBM95, and cortical astrocytes cells were used. In Fig. 5 is displayed a typical astrocyte cell stained for phaloidin-FITC, in green, (A) and for glial fibrillary acidic protein (GFAP), in red (B). Figs. 5C and 5D represent the F-actin cytoskeleton of U-87 MG and GBM95 respectively, stained for phaloidin-FITC. Values of the tether force,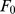, for each of the cell types are shown in Fig. 5E. We did not observe any statistically significant difference in tether force values (p>0.05 in t-test). The values found were of the order of 32 pN of force. We also measured the tether radius for each of the cell types. Fig. 5F shows the mean values measured for the tether radius of each of them. We also did not observe a statistically significant difference for the values obtained (p>0.05 in t-test). The values found were of the order of 50 nm of radius. Images of tethers extracted from astrocytes with the same stains as in (A-B), are shown in Figs. 5G and 5H, merged in Fig. 5I. They show that the tethers contain F-actin.

### PMV Induction

As tethers extracted from a cell surface always contain F-actin inside, we investigated whether it would be possible to separate the membrane from this actin cortex, in order to determine the elastic properties of pure cell membrane. As was mentioned in the Introduction, our measurement procedure would yield results contaminated by internal bilayer friction if applied to blebs. We adopted a different approach, based on inducing the formation of plasma membrane vesicles (PMV).

PMV’s (cf. Materials and Methods) are vesicles formed upon exposure of cells to low concentrations of specific reagents, which are regarded as having the general characteristics of plasma membranes. All cell types used in this work were induced to form PMV following the protocol adapted from [26]; see Materials and Methods for more details.

**Figure 6:**
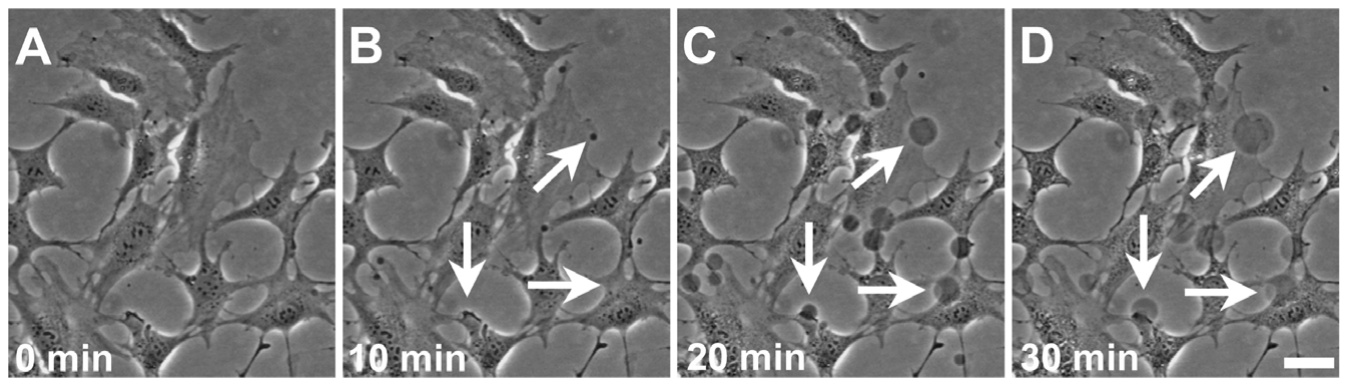
Plasma membrane vesicles growth dynamics. (A) Time 0min, U-87 MG cells immediately after treating with the PMV solution. (B) Time 10 min, appearance of small PMVs, indicated with white arrows. (C) Time 20 min, PMVs growth. (D) Time 30 min, some PMVs reach their maximum size. Scale bar is 50 µm.

Fig. 6 is a panel of images showing the time evolution of PMV formation from U-87 MG cells: just after the solution incubation (Fig. 6A), 10 min after (Fig. 6B), 20 min after (Fig. 6C) and 30 min after (Fig. 6D). It illustrates the growth of PMV’s on the cell surface, at locations indicated by white arrows.

### Tethers Extracted from PMVs

**Figure 7:**
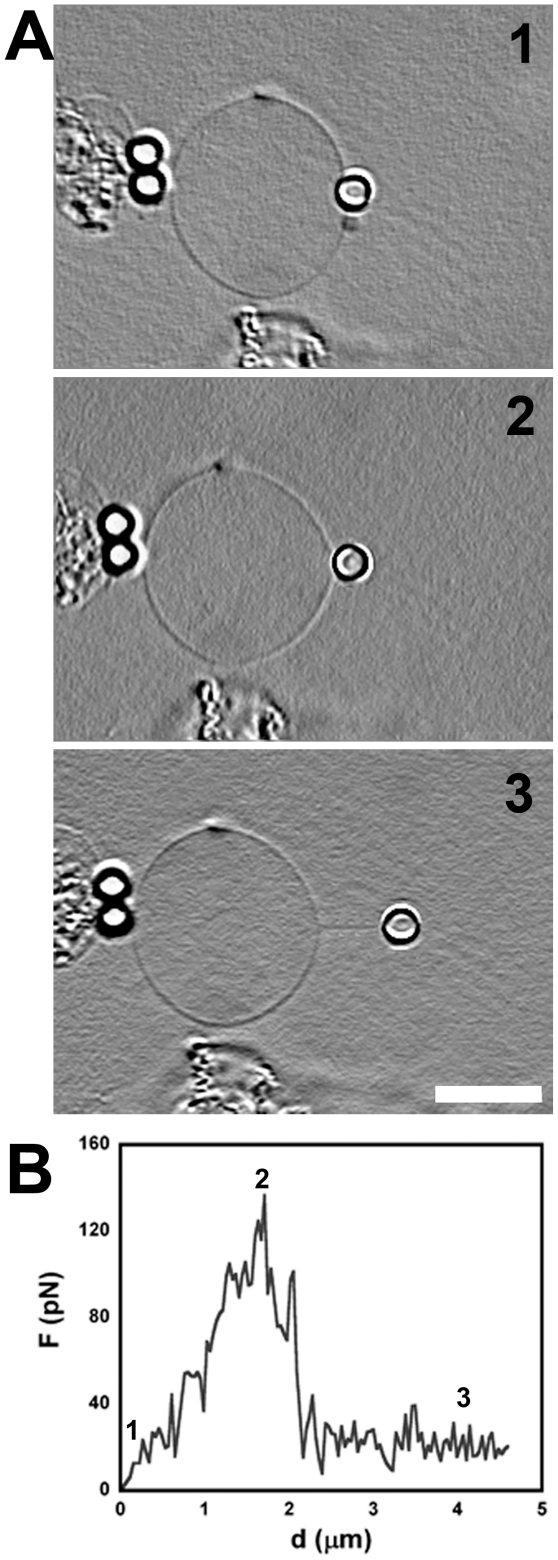
Membrane tether extraction from PMV. (A) Selection of images of the tether extraction experiment from a PMV surface. (1) Initial moment, bead is being pressed against the PMV with the optical trap, (2) moment when the force reaches the maximum value and, (3) membrane tether from PMV already formed. Scale bar is 10 µm. (B) Force curve of a tether extraction from PMV. 1, 2, and 3 represent points in the plot from the frames in (A).

Results from the extraction of a typical tether from the surface of a PMV are shown in Fig. 7. Fig. 7A shows three different situations, (1) when the bead is attached to the PMV surface, (2) when the force is the maximum force, 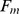, and (3) when the tether is formed and the force measured is the steady-state tether force, 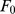. All images are in bright field, with Image J shadow north processing filter to enhance the tether image. Fig. 7B is the force × deformation curve for the tether extraction from the PMV, where the numbered points correspond to the numbered images in Fig. 7A.

**Figure 8:**
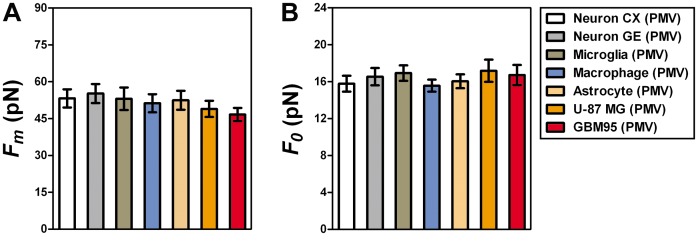
*F_m_* and *F_0_* values obtained from PMV. (A) Mean values of *F_m_* found for PMV in each of the cell types studied. (B) Mean values of *F_0_* found for PMV in each of the cell types studied. Standard errors were used as error bars. At least 20 different experiments were performed for each situation. Colors in the plots represent the different cell types used, as indicated in the legend.

Fig. 8 represents the mean maximum forces, 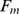
(Fig. 8A) and the mean tether forces, 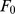 (Fig. 8B) for tether extractions from PMV of all different cell types. We did not observe any statistically significant difference either among tether force values or among maximum force values. The values found were of the order of 16 pN for *F_0_* and 55 pN for *F_m_*.

### Measurements of the Radius of Tethers Extracted from PMVs

By scrolling the frames in the video recording of the tether extraction experiments, we picked the frame that appeared to best represent the situation when the maximum force *F_m_* was reached. At this point, we measured the bead/membrane contact patch radius, *R_p_*, by image analysis.

**Figure 9:**
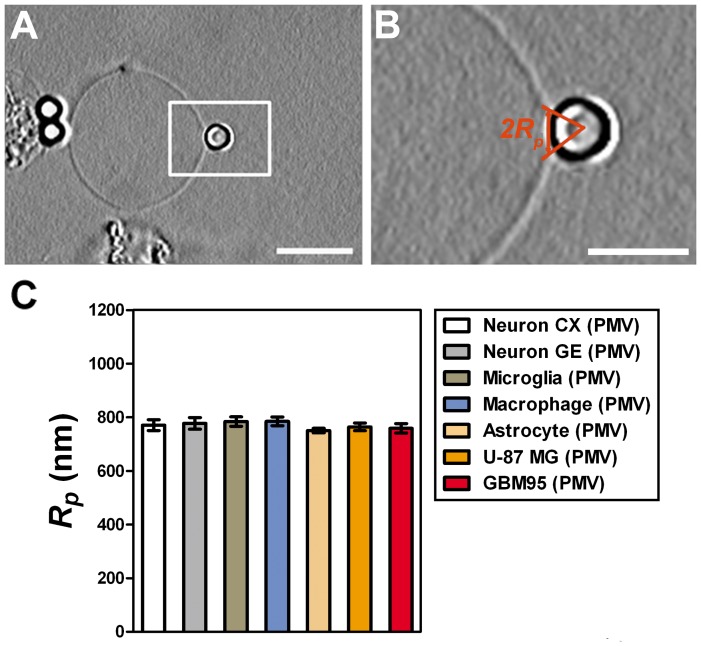
Patch radius 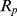 measured from the bead/membrane contact in PMV. (A–B) representative image (A) and zoom (B) indicating how *R_p_* is obtained. Scale bar for A is 10 µm and for B is 5 µm. (C) Plot of the mean values of *R_p_* for each of the cell types used. Standard errors were used as error bars. At least 20 different experiments were performed for each situation. Colors in the plots represent the different cell types used, as indicated by the legend.

Fig. 9A represents the chosen frame. Fig. 9B is a zoom of the white rectangle in Fig. 9A, indicating how we measured the patch radius, 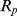. As can be seen in Fig. 9B, the bead is laterally attached. We measured 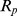 for all cell PMVs used in this work. We did not observe any statistically significant difference among 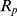 values (p>0.05 in t-test). The values found were in the range of 770 nm. This is well above the 200 nm resolution of the optical microscope.

Using the measurements of *R_p_* together with the measurements of *F_m_* and *F_0_*, we determined the value of the tether radius, *R*, for each of the PMVs used in this work, by employing Eq. (5).

### Determination of Cell Membrane Elastic Constants

Employing all the measurements of tether radius *R* and tether force *F_0_* obtained above for the surface of cells and for the PMVs, we determined, by using Eq. (4), based on the theoretical model for membrane tethers [37], [38], the surface tension and bending modulus for the membranes of all cell types studied. The results are given in Table 1.

## Discussion

It is becoming increasingly apparent that the cell membrane, interacting with the attached cytoskeleton, is an important regulator of cell function [1], [42], [43], [44], [45], [46], [47], [4].

Given the fluid character of the membrane, its surface tension is an ideal candidate for global cell signaling, in view of its fast equalization all around the cell in response to an induced change. Membrane curvature, in contrast, is locally adjustable by a variety of mechanisms and is employed for dynamic cell remodeling and movement [48], [49], [50].

These considerations emphasize the relevance of efforts to determine the parameters σ and 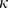 for different cell types. Tether pulling with optically trapped beads is the only direct method for such measurements [45], requiring the determination of the steady-state tether force *F_0_* and tether radius *R*. The assumption sometimes made that 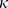 has a quasi-universal value for all cell membranes is unwarranted, as is clear from Table 1.

The values of σ and 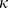 for PMV’s in Table 1 agree within roughly a factor of 2 with those for blebs in [7] and those for blebs under retraction in [22]. They also agree, to the same order of accuracy, with the values found in [9] for NIH 3T3 cells treated with cytochalasin D, a drug that disrupts actin filaments in the cytoskeleton. Thus, it is reasonable to ascribe values of these orders of magnitude to “pure cell membrane”.

The elastic constants for neurons in Table 1 are therefore close to those for pure cell membrane. This suggests a weaker interaction between membrane and the adjacent F-actin cortical cytoskeleton for neurons than for the other cell types we considered. It is also consistent with the dominant role of microtubules in neuronal function. Recent results [51] indicate that actin in axons is organized in ring-like periodic structures wrapped around the axon circumference.

Although astrocytes and glioblastoma cells share with neurons the same ectodermal origin and the same early embryonic development, they are considerably more dynamic and mobile, as was mentioned in the Introduction. Supporting the relation between membrane elastic properties and cell function, Table 1 shows that they have higher values for σ and 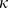. These values are very similar for both cell types. This is consistent with the finding that normal astrocytes and glioblastoma cells co-cultured with neurons show analogous interactive morphological behavior [30].

In contrast, microglia are of mesodermal origin: related to monocytes like macrophages, though “non-identical twins” [52]. In the brain parenchyma, motile amoeboid cells proliferate and differentiate into ramified microglia after reaching their definitive location [53], [35], [54]. During development, the amoeboid microglia participates in the formation of the complex network of connections in the adult brain [54], [55]. In adults, microglial ramified cells, also called resting microglia, constitute the resident macrophage population of the Central Nervous System (CNS). In vivo, the microglial processes extend over a multitude of non-overlapping territories that cover the entire neural parenchyma [56].

Studies have shown that microglial ramifications exhibit continuous movement, allowing the whole extracellular space of the CNS parenchyma to be scanned every few hours [56], [17], [57].

We see in Table 1 that resting microglia have substantially higher values of σ and 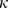 than other brain cells, values very similar to those of resting macrophages. At first sight, this might be attributed only to their different origin. However, a striking manifestation of the relationship between membrane elastic constants and cell function is the change in 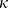 when they are activated.

For microglia, the bending modulus decreases by a factor of 3.1; for macrophages, by a factor of 3.7. The bending modulus plays, with respect to curvature, the same role as the spring constant with respect to stretching. Thus, the reduction in 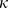 renders the membrane much softer and easier to bend, which is advantageous to engulf a foreign body. The actin remodeling and biochemical changes associated with phagocytosis are well illustrated in [16].

An analogous softening for macrophages was reported by Leporatti et al. [41]. They employed Colloidal Force Microscopy, a technique in which a microsphere glued to the cantilever of an atomic force microscope is pressed against the cell and the resulting indentation is measured against the load force. Theoretical analysis of the resulting curve yields an average value of the reduced Young’s modulus. It was found that the average Young’s modulus for LPS-stimulated macrophages is about one third of that for resting ones. Since the bending modulus is proportional to Young’s modulus, this result is consistent with our finding.

The fluorescence images in Figs. 2, 3 and 5 shows that cytoskeleton is present within tethers extracted from neurons, astrocytes and microglia, confirming our finding in [9]; see also [58]. This contradicts conventional wisdom, according to which tethers are formed by pure membrane. In all cases, the presence of actin filaments is established. For neurons, in addition, the presence of β-tubulin within tethers is also observed.

In conclusion, we have measured the elastic constants of cell membranes for all major constituent cells of the central nervous system and we have presented evidence supporting the conjectured correlation between cell membrane elasticity and the functions of these cells. We have also reconfirmed the finding in [9] that, contrary to conventional wisdom, tethers contain F-actin, as well as other cytoskeleton elements.

Existing theories of tether extraction, which we employed for the determination of the elastic constants, do not take into account the presence of cytoskeleton within the tethers. Thus, the numerical results obtained for σ and 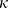 are subject to some uncertainty.

However, notwithstanding this reservation, those results are supported by several other data. As was discussed following Eq. (1), an independent determination of the surface tension, based on the maximum force *F*_max_ that the membrane can withstand around the patch radius *R_p_*, is in agreement with the obtained values. The sharp drop in the force curves beyond that point, according to the experimentally verified barrier force theory [40], [9], relates the ratio of the tether radius *R* to *R_p_* with the ratio of the tether force *F* to *F*_max_, again in agreement with the obtained values. All these radii and forces were measured. Their values, determined under uniform experimental conditions for all cell types, can be taken as data for future developments in theories of tether extraction.

Comparisons among the various cell types are also supported by independent arguments. All cells of the central nervous system, except microglia, have the same ectodermal origin and the same early embryonic development, so that they are closely related. Results for microglia, of mesodermal origin, are strikingly different, and their variation from resting to activated condition occurs within the same cell type. They are closely related to macrophage, which undergo a very similar quantitative variation, confirmed by our results ion agreement with an independent experimental technique [41].

No theory of tether extraction including cytoskeleton is yet available. In our view, an improved theory requires new experimental information about processes that take place during tether formation, including not only cortex attachment, but also the possible role of motor proteins. Work towards this aim is in progress in our laboratory.
